# Natural Language Processing–Powered Real-Time Monitoring Solution for Vaccine Sentiments and Hesitancy on Social Media: System Development and Validation

**DOI:** 10.2196/57164

**Published:** 2024-06-21

**Authors:** Liang-Chin Huang, Amanda L Eiden, Long He, Augustine Annan, Siwei Wang, Jingqi Wang, Frank J Manion, Xiaoyan Wang, Jingcheng Du, Lixia Yao

**Affiliations:** 1 Melax Tech Houston, TX United States; 2 Merck & Co, Inc Rahway, NJ United States

**Keywords:** vaccine sentiment, vaccine hesitancy, natural language processing, NLP, social media, social media platforms, real-time tracking, vaccine, vaccines, sentiment, sentiments, vaccination, vaccinations, hesitancy, attitude, attitudes, opinion, perception, perceptions, perspective, perspectives, machine learning, uptake, willing, willingness, classification

## Abstract

**Background:**

Vaccines serve as a crucial public health tool, although vaccine hesitancy continues to pose a significant threat to full vaccine uptake and, consequently, community health. Understanding and tracking vaccine hesitancy is essential for effective public health interventions; however, traditional survey methods present various limitations.

**Objective:**

This study aimed to create a real-time, natural language processing (NLP)–based tool to assess vaccine sentiment and hesitancy across 3 prominent social media platforms.

**Methods:**

We mined and curated discussions in English from Twitter (subsequently rebranded as X), Reddit, and YouTube social media platforms posted between January 1, 2011, and October 31, 2021, concerning human papillomavirus; measles, mumps, and rubella; and unspecified vaccines. We tested multiple NLP algorithms to classify vaccine sentiment into positive, neutral, or negative and to classify vaccine hesitancy using the World Health Organization’s (WHO) 3Cs (confidence, complacency, and convenience) hesitancy model, conceptualizing an online dashboard to illustrate and contextualize trends.

**Results:**

We compiled over 86 million discussions. Our top-performing NLP models displayed accuracies ranging from 0.51 to 0.78 for sentiment classification and from 0.69 to 0.91 for hesitancy classification. Explorative analysis on our platform highlighted variations in online activity about vaccine sentiment and hesitancy, suggesting unique patterns for different vaccines.

**Conclusions:**

Our innovative system performs real-time analysis of sentiment and hesitancy on 3 vaccine topics across major social networks, providing crucial trend insights to assist campaigns aimed at enhancing vaccine uptake and public health.

## Introduction

Vaccine is an essential public health intervention that has saved millions of lives and achieved a substantial global reduction in cases, hospitalizations, and health care costs associated with vaccine-preventable diseases (VPDs) [1-3]. Yet, despite their value, vaccine hesitancy persists as a barrier to full vaccine uptake. The World Health Organization (WHO) defines vaccine hesitancy as the delay or refusal of vaccination, even when vaccination services are accessible [4]. Additionally, the WHO identifies vaccine hesitancy as one of the top 10 global health threats [5]. Delay or refusal of vaccines due to vaccine hesitancy can have broad-reaching implications; unvaccinated individuals not only put themselves at risk of VPDs, such as COVID-19, but also pose a threat to the broader community or even global health [6]. This phenomenon has been documented since the advent of vaccines in over 90% of the countries [7]. Considering the case of measles, mumps, and rubella (MMR), it is crucial to uphold community protection or herd immunity, necessitating widespread vaccination to protect those unable to receive the vaccine [8]. A former London study successfully raised MMR vaccination rates from 80% to 94% in under 2 years through incentivized care packages and innovative technology use, approaching the desired herd immunity target [9].

There is a myriad of reasons for vaccine hesitancy, including personal or familial beliefs, concerns about adverse reactions or efficacy, and skepticism toward government and vaccine manufacturers [6,10-17]. This intricate web of motivations makes vaccine hesitancy a complex public health challenge [18].

Understanding vaccine hesitancy is crucial for developing effective interventions, public health education, and vaccination promotion strategies [19-22]. While surveys have traditionally served as a valuable tool for gathering public opinions on vaccination, they possess inherent limitations such as static data collection, resource intensiveness, and potential time lag [23-29]. To address these limitations, real-time tracking of vaccine hesitancy activities and trends offers public health professionals’ valuable insights. This approach helps identify critical intervention points before the vaccination uptake wanes, allowing for more targeted and timely communication efforts.

The emergence of social media platforms has enabled billions of users to engage in discussions, information sharing, and opinion expression on various subjects, including health-related topics [30]. While this presents an unprecedented opportunity for public health improvement, it also poses a significant risk linked to the dissemination of vaccine-related misinformation and disinformation [31]. Previous research has used semiautomatic methods such as manual coding and hashtag or keyword analysis to study social media vaccine discussions [32-35]. Nevertheless, these approaches may sometimes encounter potential challenges with scalability and precision. Natural language processing (NLP) is an automated method designed to effectively and accurately decipher the wealth of information in natural language text, addressing challenges such as ambiguities and probabilistic parsing, and enabling applications such as information extraction and discourse analysis [36]. This technique has emerged as a promising solution, holding the potential to mitigate these challenges and improve the precision of vaccine-related public sentiment analysis [37,38].

To address these challenges, this study’s principal aim was to create an NLP system for real-time monitoring of vaccine sentiment and hesitancy across English-language social media platforms targeting the US market. Our 3-fold contributions are (1) developing one of the first real-time monitoring systems for social media vaccine discussions that covers 3 major social media platforms and 3 vaccine topic groups [39]; (2) comprehensively evaluating multiple machine learning–based NLP models for social media post classification tasks, thus establishing a benchmark for future research; and (3) analyzing decade-long trends of sentiment and hesitancy and linked real-world events to corresponding points on the trends for multiple vaccine targets.

## Methods

### Overview

**Figure 1:**
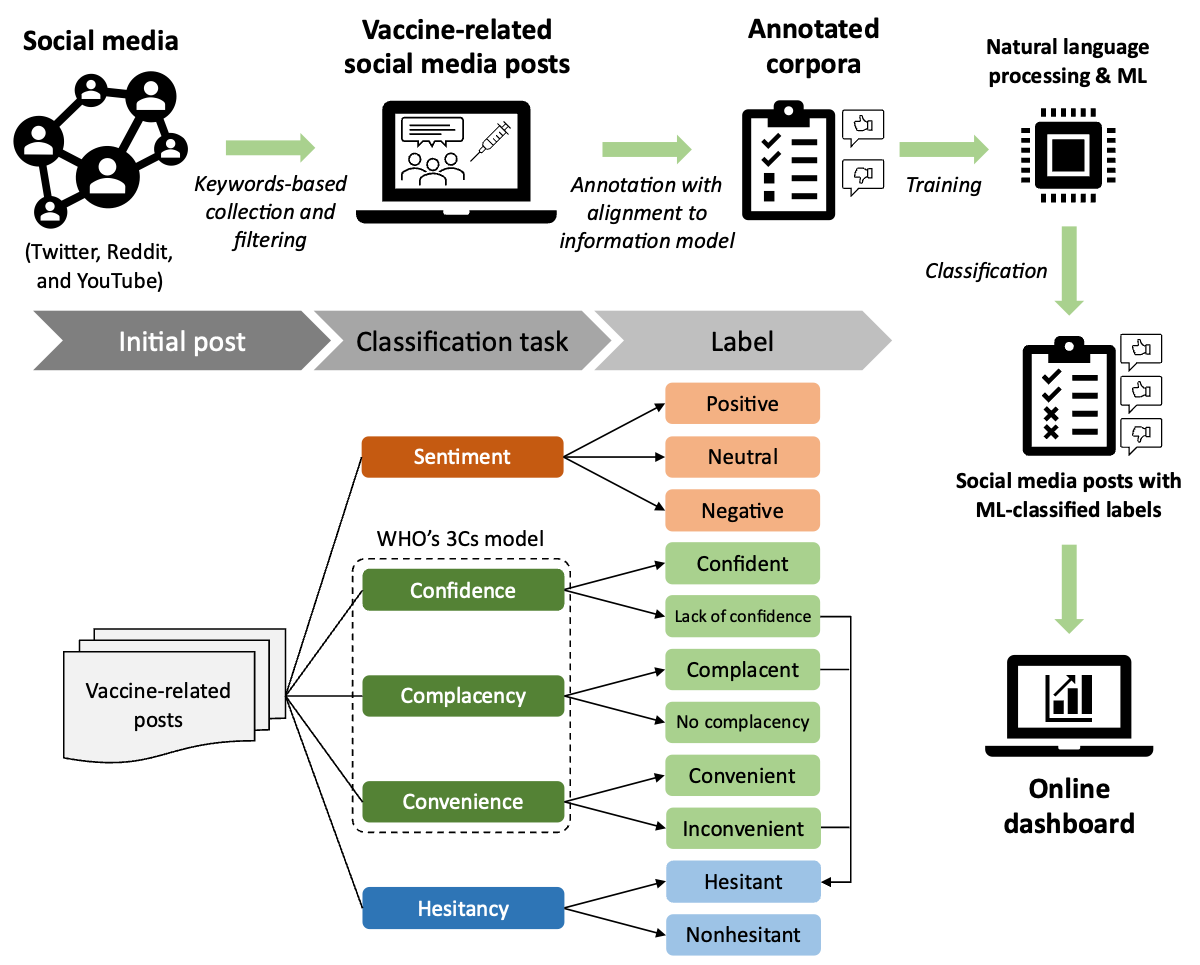
The overview of study design and classifications used to evaluate vaccine-related posts. 3Cs: confidence, complacency, and convenience; ML: machine learning; WHO: World Health Organization.

We followed a systematic approach to monitor vaccine sentiment and hesitancy posts on Twitter (subsequently rebranded as X), Reddit, and YouTube. We selected Twitter, Reddit, and YouTube as they are the primary social media platforms offering substantial volumes of posts through application programming interface (API) access [40-43]. We focused exclusively on English language posts given the widespread use of English in the largest market countries for our target vaccines and with regard to the accessibility of English language social media. Other platforms and languages, such as Facebook and Spanish [44], may be of interest for future studies; however, these served as a first approach to research. Figure 1 illustrates our workflow, including data annotation, NLP algorithms, and an online dashboard.

First, we categorized vaccine sentiment into positive, negative, and neutral, which were the labels also used in other sentiment analyses using social media data [45,46]. Then, we aligned vaccine hesitancy with the WHO’s 3Cs (confidence, complacency, and convenience) vaccine hesitancy model, described in further detail in the *3Cs Vaccine Hesitancy Annotation* section [4]. The definitions of post sentiment and vaccine hesitancy are comprehensively presented in Table 1. We collected data using vaccine-specific search queries (see Table S1 in Multimedia Appendix 1) for relevant posts from the 3 social media platforms. To ensure the quality and reliability of the data, we collaborated with medical experts to create annotated corpora aligned with the information model. These corpora were then used to train NLP algorithms to automatically extract vaccine sentiment and hesitancy content. Finally, we developed an online dashboard to provide real-time insights into vaccine sentiment and hesitancy trends. Our study focuses on evaluating the vaccine sentiment and hesitancy of human papillomavirus (HPV), MMR, and general or unspecified vaccines. The critical role of the vaccines is exemplified by the HPV vaccine, which has effectively reduced prevalent HPV infections and precancerous lesions, underlining the importance of global implementation [47], and the MMR vaccine is renowned for its safety and efficacy, which has greatly mitigated endemic diseases in the United States [48]. Despite these successes, challenges such as insufficient vaccination coverage, increasing hesitancy, and the resurgence of mumps, attributed to waning immunity and antigenic variation, persist worldwide. Throughout the COVID-19 pandemic up to 2022, HPV and MMR were the vaccines that maintained the greatest negative impact on routine vaccinations in the United States, suggesting a need for proactive efforts to increase vaccination coverage to prevent associated health complications and costs [49].

**Table 1:** Definitions of post sentiment and hesitancy.

Classification task and label		Definition
**Sentiment**		
	Positive	Posts that mention, report, or share positive news, opinions, or stories about vaccines or vaccination.
	Neutral	Posts that are related to vaccines or vaccination topics but contain no sentiment, the sentiment is unclear, or they contain both negative and positive sentiments.
	Negative	Posts that mention, report, or share negative news, opinions, or stories about vaccines or vaccination, which may discourage vaccination.
**Confidence**		
	Confident	Posts reflecting a trust in the effectiveness and safety of vaccines, the vaccine delivery system, or policy makers’ motivations.
	Lack of confidence	Posts reflecting a lack of trust in the effectiveness and safety of vaccines, the vaccine delivery system, or policy makers’ motivations.
**Complacency**		
	Complacent	Posts where the perceived risks of VPDs^a^ are low and vaccination is deemed as an unnecessary preventive action.
	No complacency	Posts where the perceived risks of VPDs are high and vaccination is deemed as a necessary preventive action.
**Convenience**		
	Convenient	Posts where physical availability, affordability and willingness to pay, geographical accessibility, ability to understand (language and health literacy), and appeal of immunization services do not affect uptake.
	Inconvenient	Posts where physical availability, affordability and willingness to pay, geographical accessibility, ability to understand (language and health literacy), and appeal of immunization services affect uptake.
**Hesitancy**		
	Hesitant	The post is labeled as lack of confidence, complacent, or inconvenient.
	Nonhesitant	The post is not labeled as lack of confidence, complacent, or inconvenient.

^a^VPD: vaccine-preventable disease.

### Social Media Data Collection

The systematic collection of social media data spanned from January 1, 2011, to October 31, 2021, across 3 platforms—Twitter, Reddit, and YouTube. During initial exploratory analysis, we recognized variations in text nature and query logic across these platforms, leading us to tailor our search queries for each platform to collect relevant posts while excluding irrelevant ones. Table S1 in Multimedia Appendix 1 lists the customized queries on each platform for each vaccine topic group, which include both inclusion and exclusion keywords. We retrieved the results (relevant posts) using the APIs provided by the 3 platforms. Details about the software versions are described in the Multimedia Appendix 1. To clarify ethical considerations and data privacy issues, when gathering data from Twitter, YouTube, and Reddit, we adhered to their API’s data privacy policies and ensured the deidentification of all posts and videos by assigning them a unique random ID.

### Ethical Considerations

Ethics board review was not required, as all modelling data came from public sources and there were no ethical issues. The data privacy policies of the application program interfaces (APIs) of Twitter, YouTube, and Reddit were followed when gathering data. We ensured the deidentification of all posts and videos by assigning them a unique random ID.

### Data Annotation

From the retrieved results, approximately 90 million posts, we randomly selected 60,000 social media discussions. These posts were manually annotated to build both training and evaluation data sets, which were used for building the text classifiers. We selected 20,000 posts for annotation, including 10,000 tweets, 5000 Reddit posts, and 5000 YouTube comments for each vaccine topic group, including HPV vaccine, MMR vaccine, and general or unspecified vaccines. During annotator training, 4 annotators with a medical training background were recruited for the annotation. An annotation guideline was developed. All annotators first annotated the same 1000 tweets, 1000 Reddit posts, and 1000 YouTube posts independently, and then discussed collectively for any discrepancies. After all discrepancies were resolved through discussions, these annotators began to annotate the rest of the social media posts. A 2-fold annotation strategy was used, where first, we annotated the sentiment of the post as positive, neutral, or negative, assigning only 1 category to each post; and second, we annotated vaccine hesitancy based on the constructs of the WHO 3Cs model, which include confidence, complacency, and convenience (Figure 1). These annotation categories also define each classification task.

### Sentiment Annotation

The annotation task involved assigning 1 of 3 sentiment labels to each post, which constituted a multiple-class classification problem. The labels and corresponding illustrative examples are defined in Textbox 1.

**Definitions and examples of sentiment labels.**

- Positive: posts that mention, report, or share positive news, opinions, or stories about vaccines or vaccination.
  Example: “HPV vaccine, prevents against the two HPV types, 16 and 18, which cause 70% of cervical cancers”
  Example: “Get vaccinated against HPV to protect you in the future for now!”
- Neutral: posts that are related to vaccines or vaccination topics but contain no sentiment, the sentiment is unclear, or they contain both negative and positive sentiments.
  Example: “The following report is specifically for the MMR vaccine, but you can browse around for others”
  Example: “I just learned that there are more than 50 strains of HPV...I always thought the vaccine prevented all strains.”
- Negative: posts that mention, report, or share negative news, opinions, or stories about vaccines or vaccination, which may discourage vaccination.
  Example: “According to a report, thousands of kids suffer permanent injury or death by getting vaccines”
  Example: “Believe it? Vaccines have killed 1000 more kids than any measles!”

### 3Cs Vaccine Hesitancy Annotation

The annotation task involved assigning multiple labels to each post according to the 3Cs model constructs. Annotators checked each construct to determine whether the post was related to it separately. If any of the constructs were labeled as “lack of confidence,” “complacent,” or “inconvenient,” we considered the post as vaccine hesitant; otherwise, it was considered vaccine nonhesitant. Definitions and examples for each 3Cs model construct are provided in Textbox 2.

Table S2 in Multimedia Appendix 1 provides examples of specific social media posts with annotations for the different categories. The distribution of annotated posts in each sentiment and 3Cs construct for each platform and vaccine topic group is shown in Table S3 in Multimedia Appendix 1.

**Definitions and examples of World Health Organization’s 3Cs (confidence, complacency, and convenience) model.**

- Lack of confidence: posts reflecting a lack of trust in the effectiveness and safety of vaccines, the vaccine delivery system, or policy makers’ motivations.
  Example: “Fully vaccinated are 30 times more likely to get COVID-19, and 10 times more likely to require hospitalization.”
  Example: “The vaccine label includes all these events. Concerns have been raised about reports of deaths occurring in individuals after receiving that vaccine.”
- Complacency: posts where the perceived risks of vaccine-preventable diseases are low, and vaccination is deemed as an unnecessary preventive action.
  Example: “Why do adults need to know about the measles vaccine? The measles is a benign disease and there is no need for vaccines.”
  Example: “I wasn’t vaccinated against a preventable disease. It’s not always just a life-or-death dichotomy - I recovered.”
- Inconvenience or convenience: posts where physical availability, affordability and willingness to pay, geographical accessibility, ability to understand (language and health literacy), and appeal of immunization services affect uptake.
- Example: “I am 30-year-old man and am looking for an HPV vaccine. Unfortunately, my insurance only covers it for women. I am particularly at risk for certain cancers. I really don’t understand how insurance companies are allowed to make the gender distinction when the FDA approved it for both.”

### Text Classification Algorithms

#### Overview

To classify the sentiment and hesitancy of social media posts, we compared the performance of 5 text classification algorithms—logistic regression (LR) [50], support vector machine (SVM) [51], random forest [52], extreme gradient boosting (XGBoost) [53], and Snorkel [54]. Each of these models has unique characteristics, which are summarized below.

#### LR Algorithm

LR is a classic statistical methodology that models a binary dependent variable using a logistic function. It is favored in medical research due to its ability to determine the odds ratio, indicating the potential change in outcome probabilities [55].

#### SVM Algorithm

SVM is one of the most robust classification methods based on statistical learning frameworks. It finds a hyperplane in an N-dimensional space that distinctly classifies data points. In medical text mining, SVM combined with other algorithms has demonstrated effective performance in extracting and recognizing entities in clinical text, contributing notably to improved patient care [56].

#### Random Forest Algorithm

Random forest is a classifier that uses ensemble learning to combine decision tree classifiers through bagging or bootstrap aggregating. It has been applied to highly ranked features obtained through suitable ranker algorithms and has shown promising results in medical data classification tasks, enhancing the prediction accuracy for various diseases [57].

#### XGBoost Algorithm

XGBoost is an ensemble of algorithms that turn weak learners into strong learners by focusing on where the individual models went wrong. In gradient boosting, individual weak models train upon the difference between the classification and the actual results. It has been effective in mining and classifying suggestive sentences from online customer reviews by combining them with a word-embedding approach [58].

#### Snorkel Algorithm

Snorkel is a system that enables users to train models without hand labeling all training data by writing their labeling functions. Using Snorkel enables the extraction of chemical reaction relationships from biomedical literature abstracts, supporting the understanding of biological processes without requiring a large, labeled training data set [59].

We extracted the term frequency–inverse document frequency vector for each word in all text classification algorithms using *scikit-learn*’s *TfidfTransformer* function with default parameter settings. Term frequency–inverse document frequency evaluates how relevant a word is to a text in a collection of texts [60]. If the model encounters a new post with words or symbols not included in its original bag of words, it will effectively ignore those words during the transformation process. To ensure a balanced training set, the 3 class-balancing methods implemented by Python *imblearn* package applied were (1) random oversampling, (2) synthetic minority over-sampling technique (SMOTE) [61], and (3) SVM-based SMOTE [62] (with the default parameter settings, specifically k_neighbors=5, as they exhibited the optimal performance within the developer’s data set [61]). SMOTE randomly selects a minority class instance, finds one of its nearest minority class neighbors, and then synthesizes an instance between these 2 instances in the feature space. SVM-based SMOTE uses support vectors to determine the decision boundaries and then synthesizes a minority class instance along the decision boundary.

### NLP Evaluation

The evaluation data sets were created from the annotated corpora and randomly divided into training, validation, and test sets in a 6:2:2 ratio to assess the performance of the 5 text classification algorithms. The models were trained on the training sets, optimized on the validation sets, and then evaluated on the test sets. The following key metrics were calculated to evaluate the models:



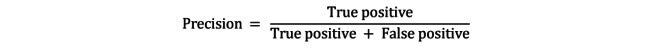





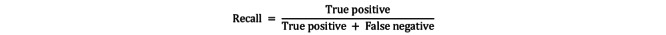





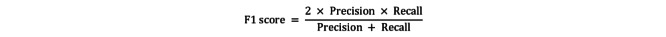





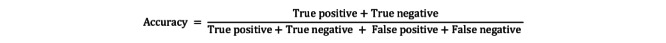



A true positive occurs when the model accurately classifies the positive class (positive, negative, or neutral for sentiment; true for 3Cs model constructs). A true negative occurs when the model accurately classifies the negative class (nonpositive, nonnegative, or nonneutral for sentiment; false for 3Cs model constructs). A false positive is an incorrect positive classification, while a false negative is an incorrect negative classification. As the sentiment and hesitancy labels in Tweets, Reddit posts, and YouTube comments are imbalanced, we optimized our models based on *F*_1_-scores, which balance precision and recall, rather than accuracy. The purpose of optimizing a model based on *F*_1_-scores when dealing with imbalanced labels is to achieve a better balance between precision and recall, thereby improving the overall performance of the model. This is especially important in imbalanced data sets where the cost of misclassification can be high.

### Dashboard Development

We designed a user-friendly, web-based visualization dashboard [39] for real-time analysis of trends in vaccine sentiment and hesitancy over time and geography (Figure S1A-C in Multimedia Appendix 1). The dashboard also allows for comparisons of sentiment and hesitancy across different social media platforms and vaccine topic groups (Figure S1D in Multimedia Appendix 1). The NLP models were optimized based on their *F*_1_-scores to address the imbalanced labels of sentiment and hesitancy in tweets, Reddit posts, and YouTube comments. The selected models are applied to all unlabeled data collected from 2011 to 2021. Technical details are described and represented in Figure S2 in Multimedia Appendix 1.

## Results

### Social Media Data Collection Summary

**Figure 2:**
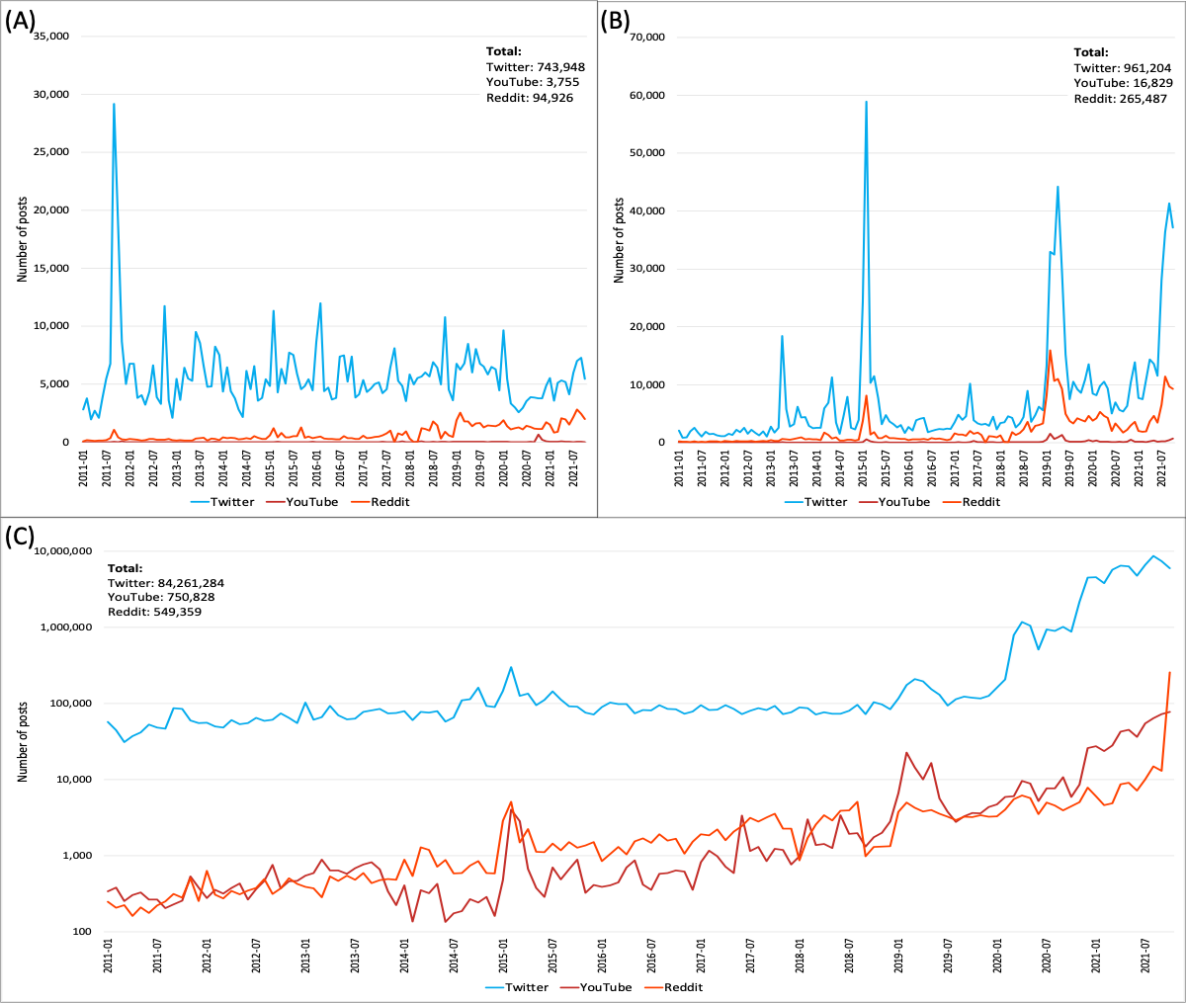
The long and short-term trends of collected vaccine-related social media post data across 3 different platforms for different vaccine topic groups: (A) human papillomavirus (HPV); (B) measles, mumps, and rubella (MMR); and (C) general or unspecified vaccine.

From January 1, 2011, to October 31, 2021, we collected 86 million posts from Twitter, 0.9 million from Reddit, and 76,000 from YouTube, which were related to vaccines. The most widely discussed topic across all 3 platforms was the general or unspecified vaccine, followed by the MMR and then HPV vaccines. We observed a substantial increase in the general vaccine-related discussions on Twitter and Reddit starting in early 2020, coinciding with the onset of the COVID-19 pandemic. The collected social media data and growth trends are plotted in Figure 2.

### NLP Performance on Vaccine Sentiment and Hesitancy

We tested all combinations of the 5 NLP algorithms. The performances in sentiment classification, hesitancy classification, and 3Cs classifications are presented in Table 2. The best-performing algorithms (according to *F*_1_-scores) and detailed performance scores for different classification tasks are shown in Tables S4-S8 in Multimedia Appendix 1. In sentiment classification, LR outperformed other algorithms in 7 out of 9 platform–vaccine topic group combinations, with overall accuracies ranging from 0.51 to 0.78 (Table S4 in Multimedia Appendix 1). The macroaveraged *F*_1_-scores of negative, neutral, and positive sentiment classifications across different platforms and vaccine topic groups were 0.43, 0.67, and 0.53, respectively. In hesitancy classification, LR outperformed other algorithms in 6 platform–vaccine topic group combinations, with overall accuracies ranging from 0.69 to 0.91 (Table S5 in Multimedia Appendix 1). The macroaveraged *F*_1_-scores of nonhesitancy and hesitancy classifications were 0.86 and 0.40, respectively. Notably, Reddit users had fewer negative sentiment posts, resulting in lower performance in classifying negative sentiment. In addition, as Reddit had fewer hesitancy posts, classifying hesitancy was more challenging than on Twitter and YouTube.

Our evaluation of various algorithms and class-balancing methods for each platform and vaccine topic group revealed that Snorkel performed best in 3 platform–vaccine topic group combinations in vaccine hesitancy classifications, with overall accuracies ranging from 0.69 to 0.98 (Table S6 in Multimedia Appendix 1). The macroaveraged *F*_1_-scores for lack of confidence and nonlack of confidence classifications were 0.88 and 0.45, respectively. Similarly, for complacency classifications, Snorkel outperformed other algorithms in 4 platform–vaccine topic group combinations, with overall accuracies ranging from 0.64 to 0.99 (Table S7 in Multimedia Appendix 1). The macroaveraged *F*_1_-scores for noncomplacency and complacency classifications were 0.89 and 0.49, respectively. Inconvenience classifications were significantly improved with Snorkel in 8 platform–vaccine topic group combinations, with overall accuracies ranging from 0.89 to 0.99 (Table S8 in Multimedia Appendix 1). However, the results are biased as there were limited posts with convenience information on all 3 social media platforms, which may impact generalizability. The macroaveraged *F*_1_-scores for noninconvenience and inconvenience classifications were 0.98 and 0.38, respectively. Our findings demonstrate that advanced text classification algorithms such as XGBoost and Snorkel outperformed other algorithms in highly class-imbalanced situations, even when different class-balancing methods were applied.

We have created a web-based dashboard building upon those best-performing NLP algorithms to extract vaccine sentiment and hesitancy from social media posts. The dashboard summarizes posts from the 3 social media platforms and allows users to analyze temporal trends and geographic clustering easily. It offers different views, including 3 social media platform–centric views and a comparison view that enables users to compare selected vaccine topic groups and sentiment or hesitancy (Figure S1 in Multimedia Appendix 1).

**Figure 3:**
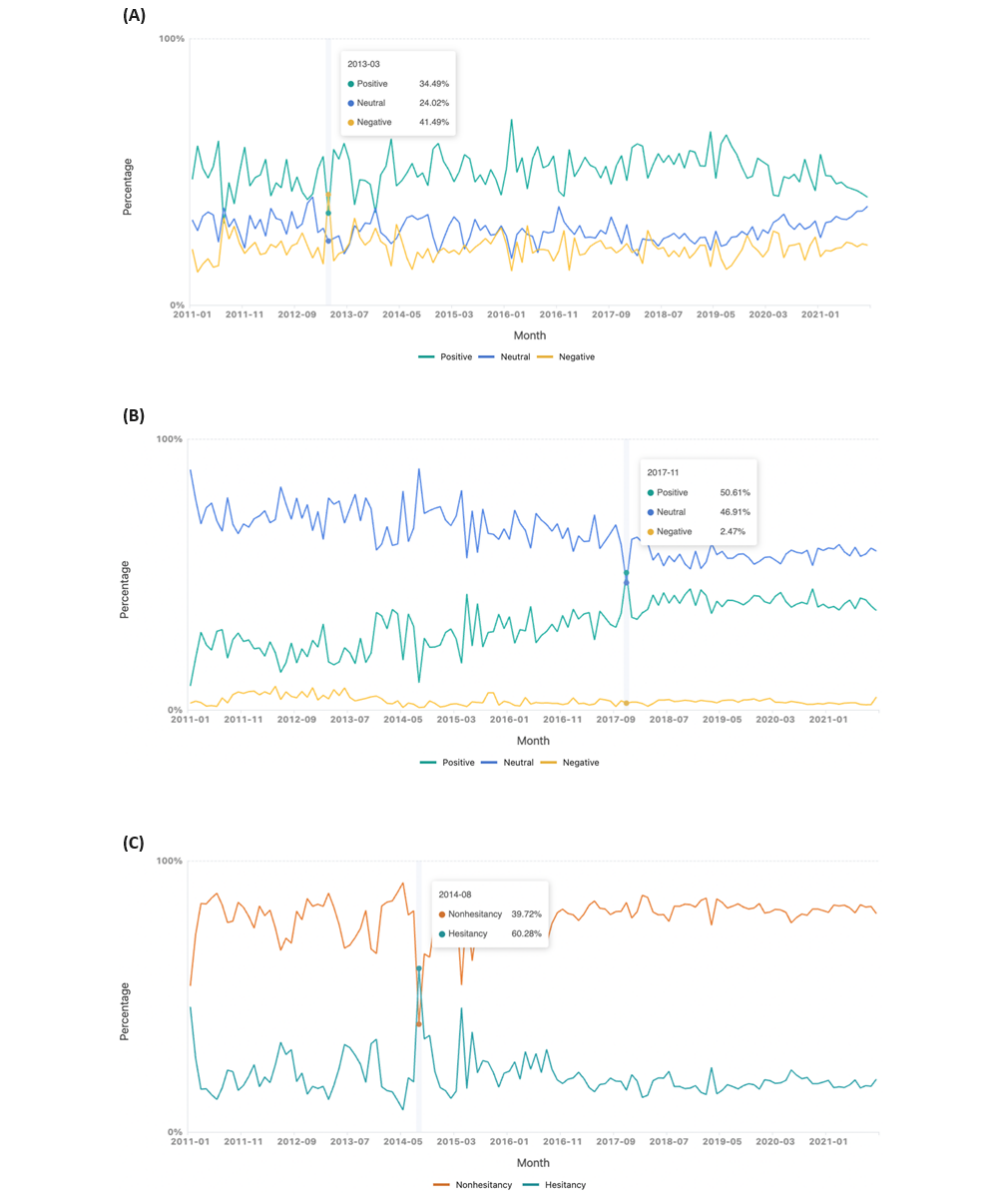
Temporal trends of vaccine sentiment and hesitancy. (A) Aggregation of 3 social media platform data sources to evaluate vaccine sentiment for HPV vaccine–related posts. (B) Comparison of vaccine sentiment for MMR vaccines. (C) Comparison of vaccine hesitancy for MMR vaccine. HPV: human papillomavirus; MMR: measles, mumps, and rubella.

When analyzing the sentiment of HPV vaccine posts across 3 social media platforms from January 2011 to October 2021 (Figure 3A), we observed that the ratio of positive sentiment was generally higher than that of neutral and negative sentiment. We also compared vaccine sentiment across 3 social media platforms for MMR vaccines from January 2011 to October 2021 (Figure 3B). Overall, posts expressed positive sentiment toward MMR, with most being neutral. Taking the hesitancy of MMR vaccine as an example, the overall trend shows that the social media posts across 3 social media platforms have a higher ratio of nonhesitancy than hesitancy (Figure 3C).

**Table 2:** NLP^a^ performance (measured by F1-scores and accuracy) on vaccine sentiment and hesitancy.

Performance		Twitter			Reddit				YouTube		
		HPV^b^	MMR^c^	General^d^	HPV	MMR	General	HPV		MMR	General
**Sentiment**											
	Positive *F*_1_-score	0.87	0.57	0.47	0.67	0.50	0.35	0.58		0.53	0.19
	Neutral *F*_1_-score	0.71	0.67	0.83	0.67	0.65	0.86	0.51		0.59	0.51
	Negative *F*_1_-score	0.41	0.53	0.43	0.32	0.26	0.21	0.60		0.49	0.59
	Accuracy	0.78	0.61	0.73	0.63	0.55	0.75	0.56		0.55	0.51
**Confidence**											
	Confident *F*_1_-score	0.35	0.31	0.52	0.35	0.62	0.56	0.44		0.29	0.63
	Lack of confidence *F*_1_-score	0.88	0.95	0.79	0.86	0.74	0.84	0.89		0.99	0.98
	Accuracy	0.80	0.90	0.71	0.77	0.69	0.77	0.82		0.98	0.95
**Complacency**											
	Complacent *F*_1_-score	0.47	0.36	0.41	0.43	0.68	0.60	0.33		0.50	0.60
	No complacency *F*_1_-score	0.94	0.91	0.81	0.93	0.59	0.96	0.91		1.00	0.97
	Accuracy	0.89	0.84	0.71	0.88	0.64	0.93	0.84		0.99	0.95
**Convenience**											
	Convenient *F*_1_-score	0.96	0.99	0.99	0.94	0.95	0.98	0.98		1.00	0.99
	Inconvenient *F*_1_-score	0.48	0.18	0.55	0.67	0.17	0.50	0.17		0.50	0.20
	Accuracy	0.92	0.98	0.98	0.89	0.91	0.97	0.96		0.99	0.98
**Hesitancy**											
	Hesitant *F*_1_-score	0.40	0.44	0.38	0.19	0.23	0.20	0.58		0.53	0.61
	Nonhesitant *F*_1_-score	0.94	0.90	0.89	0.87	0.81	0.95	0.81		0.83	0.76
	Accuracy	0.90	0.83	0.82	0.78	0.69	0.91	0.73		0.75	0.70

^a^NLP: natural language processing.

^b^HPV: human papillomavirus.

^c^MMR: measles, mumps, and rubella.

^d^General: general or unspecified vaccines.

## Discussion

### Principal Findings

Our analysis of temporal trends in vaccine-related sentiment on social media platforms yielded valuable insights into the dynamics of public perception. A total of 5 different classification algorithms were subjected to tests for performance in sentiment and hesitancy classifications, revealing that advanced text classification algorithms such as XGBoost and Snorkel outperformed others in classifying hesitancy, complacency, and other factors, while LR had a superior performance for sentiment classification. The superior performance of LR could potentially be attributed to its enhanced ability to effectively handle binary classification challenges and manage noise variables [63]. As the use of artificial intelligence platforms is increasingly becoming accessible for public use, it is crucial to gain an understanding of their accuracy and limitations. Traditional machine learning algorithms have the ability to predict outcomes but often lack transparency. Hence, enhancing public understanding and advancing toward explainable artificial intelligence is vital for error rectification and improved model efficacy for social media research [64].

When evaluating trends for the HPV vaccine, overall positive sentiment outweighed neutral and negative sentiment (Figure 3A), a notable exception occurred in March 2013. During this period, posts with negative sentiment on all 3 platforms surpassed those with 34% (2270/6582) positive and 24% (1581/6582) neutral sentiment, constituting 41% (2731/6582) of the total. This spike in negative sentiment can be attributed to news articles published in March 2013; for example, “Worried Parents Balk At HPV Vaccine For Daughters” by National Public Radio [65] and “Side Effect Fears Stop Parents from Getting HPV Vaccine for Daughters” by CBS News [66]. These articles highlighted concerns and fears about the HPV vaccine. Afterward, specific studies were conducted and published to further investigate these concerns and fears [67,68]. Notably, the HPV vaccines have been found to be safe in several studies and strongly recommended by the Centers for Disease Control and Prevention (CDC), etc [69,70].

Conversely, overall, posts expressed more neutral sentiment toward MMR than positive sentiment (Figure 3B), with an exception in November 2017. During this month, 51% (2844/5619) of posts expressed positive sentiment and 47% (2636/5619) were neutral. We found that a mumps outbreak was observed right before November 2017, which may have encouraged people to discuss the importance of MMR vaccination. News articles highlighted this outbreak, for example, “Third dose of mumps vaccine could help stop outbreaks, researchers say” by PBS News Hour [71] and “CDC recommends booster shot of MMR vaccine during mumps outbreaks” by CNN [72] mentioned the outbreak and recommended the booster shot of MMR vaccine.

When tracking vaccine hesitancy, we found that the social media posts with a higher ratio of hesitancy were only observed in August 2014 (Figure 3C). During this month, some examples of articles could be associated with vaccine hesitancy: “Journal questions validity of autism and vaccine study” by CNN [73] and “Whistleblower Claims CDC Covered Up Data Showing Vaccine-Autism Link” by TIME [74]. While speculation, particularly among antivaccination subpopulations, continues to surround the discredited study linking MMR vaccines with autism, it is crucial to emphasize that this link has been unequivocally debunked by subsequent research, and organizations such as the CDC and WHO have clarified that no such association exists [75-77]. Nonetheless, these news articles, considered by some as antivaccine propaganda, may partially explain the observed trends in MMR vaccine hesitancy during August 2014.

### Strengths and Limitations

In this study, we introduced an NLP-powered online monitoring tool for tracking vaccine-related discussions on multiple social media platforms, covering 3 vaccine topic groups. Our system provides several features that distinguish it from existing tools. It uses NLP algorithms to perform sentiment analysis on social media posts and facilitates the tracking of temporal trends and geographic clustering of vaccine sentiment and hesitancy through visualization. In addition, our system enables users to compare vaccine sentiment and hesitancy across different social media platforms. We have publicly shared our annotated social media vaccine corpora, and we have evaluated several text classification algorithms, providing a benchmark for future research. One of the hypothetical use cases is that our NLP-based tool’s application spans from gauging vaccine sentiment during disease outbreaks to when a new vaccine is introduced. During an outbreak, the tool effectively analyzed sentiments toward measles vaccination, facilitating adjustments in public health campaigns.

While our proposed method uses the coarse-grained sentiment model (ie, represents the sentiment as a positive or negative class), fine-grained sentiment models, unlike traditional independent dimensional approaches, beneficially incorporate relations between dimensions, such as valence and arousal, into deep neural networks, thereby providing more nuanced, real-valued sentiment analysis and enhancing prediction accuracy [78-81]. These models prove particularly valuable in language-specific applications and are capable of classifying emotion categories and simultaneously predicting valence, arousal, and dominance scores for specific sentences, providing more nuanced sentiment analysis compared with simple positive or negative classifications.

Beyond the limitations inherent in the sentiment model, our approach also encounters constraints due to the use of traditional machine learning algorithms. Deep learning methods for word or sentiment embedding offer enhanced performance in sentiment analysis tasks by integrating external knowledge such as sentiment polarity and emotional semantics into word vectors [82-87]. They leverage neural networks and multitask learning to create task-specific embeddings, improving the accuracy of tasks such as sentiment and emotion analysis and sarcasm and stress detection [82-84,86]. Furthermore, these methods can adapt to the dynamic nature of language, handling out-of-vocabulary words and context-specific word meanings, proving more accurate and comprehensive than traditional word embeddings [86,87]. In future iterations, we plan to enrich our tool by integrating cutting-edge methods, alongside a more robust evaluation method such as time series cross-validation [88].

While previous studies have used NLP for sentiment analysis on COVID-19 vaccination and information exposure analysis regarding the HPV vaccine using Twitter data sets [40,89], and have investigated the temporal and geographic variations in public perceptions of the HPV vaccine [90], our tool extends its functionality to include a broader spectrum of platforms for tracking different vaccine sentiment and hesitancy on social media. Despite the scientific evidence supporting the safety and efficacy of vaccines, vaccine hesitancy sentiments on social media can impact public confidence regarding vaccination [91]. Our tool is designed to quickly identify surges in vaccine hesitancy and thereby could be a tool to assist public health professionals in responding promptly with accurate information and effective vaccine promotion strategies.

However, it is essential to acknowledge the inherent limitations of using social media as a public health surveillance tool. These limitations include geography and language restrictions, as well as potential population, age, and gender biases, given that social media users may not represent the general population [92-94]. The user diversity across various social media platforms might partly account for the variation in sentiment and hesitancy label distributions. For example, YouTube has a high volume of users, but Twitter had the most activity in our study because people may view YouTube videos without leaving comments [93]. Moreover, owners of YouTube channels also have the option to disable comments on their uploaded videos. In addition, YouTube comments are highly tied to the content of the videos that the model might not have access to, leading to misinterpretations of sentiment and hesitancy. These biases and variabilities could partly account for the lower prediction accuracy observed for YouTube. Therefore, caution should be exercised when interpreting findings based on social media data, particularly considering the varying distributions of sentiment and hesitancy across different social media platforms in our study. Another limitation pertains to the absence of a weighting system in the dashboard. Currently, the impact of each post, considering variables such as the number of views or reposts, is not considered. In addition, private interactions, specifically on sites such as Facebook, might go unnoticed and this lack of access to private dialogues could limit the comprehensiveness of the responses we capture. Finally, there is the possibility of shifts in user behavior to emerging social media platforms, such as TikTok, introducing additional population bias if such platforms are not included in further analyses.

### Conclusions

This study successfully developed an innovative real-time monitoring system for analyzing vaccine sentiment and hesitancy across 3 major social media platforms. This system uses NLP and machine learning to mine and classify social media discussions on vaccines, providing valuable insights into public sentiment and hesitancy trends. The application of this tool presents significant implications for public health strategies, aiding in promptly identifying and mitigating vaccine misinformation, enhancing vaccine uptake, and assisting in the execution of targeted health campaigns. Moreover, it encourages health care professionals to foster an evidence-based discourse around vaccines, thus counteracting misinformation and improving public health outcomes.

## Appendix

Multimedia Appendix 1 The online dashboard's user interface, architecture, and performances.

## Abbreviations

3Cs: confidence, complacency, and convenience
API: application programming interface
CDC: Centers for Disease Control and Prevention
HPV: human papillomavirus
LR: logistic regression
MMR: measles, mumps, and rubella
NLP: natural language processing
SMOTE: synthetic minority over-sampling technique
SVM: support vector machine
VPD: vaccine-preventable disease
WHO: World Health Organization
XGBoost: extreme gradient boosting
