# Supplementary material for: Natural Language Processing–Powered Real-Time Monitoring Solution for Vaccine Sentiments and Hesitancy on Social Media: System Development and Validation
Source: JMIR Med Inform. 2024 Jun 21;12:e57164. doi: 10.2196/57164 (PMC11226933; doi:10.2196/57164)
Supplement: Multimedia Appendix 1 [file medinform_v12i1e57164_app1.docx]

### Social Media Data Collection and Dashboard

We retrieved the results (relevant posts) using the application programming interface (APIs) provided by the three platforms. We utilized various software for the data collection process from social media platforms. For Twitter data, the initial collection was performed using Tweepy version 4.8.0 at no cost; however, this method is no longer effective due to API changes. In response to these developments, we have created proprietary code to collect data using the Twitter Enterprise Search API, an approach that significantly reduces the limitations imposed by a daily cap, allowing the download of over 50 million Tweets per month. For Reddit data, we initially used pmaw version 2.1.3, also free, but like Twitter, this method has become defunct due to API modifications. Lastly, for YouTube data, we employed google-api-python-client version 2.50.0, which remains functional at the time of writing. The dashboard’s front-end user interface was created using React.js, while the back-end infrastructure was built using Java SpringBoot, following a typical layered architecture. Figure S2 provides an illustration of the data collection, processing, and storage pipeline. Social media posts are collected by the data collector module, then passed to the data cleaner module for filtering and de-identification. The cleaned posts are stored in Amazon S3 storage. The best text classifiers were used to classify the sentiment and hesitancy of the posts for each vaccine topic group and platform, and the resulting labels were stored in AWS OpenSearch.


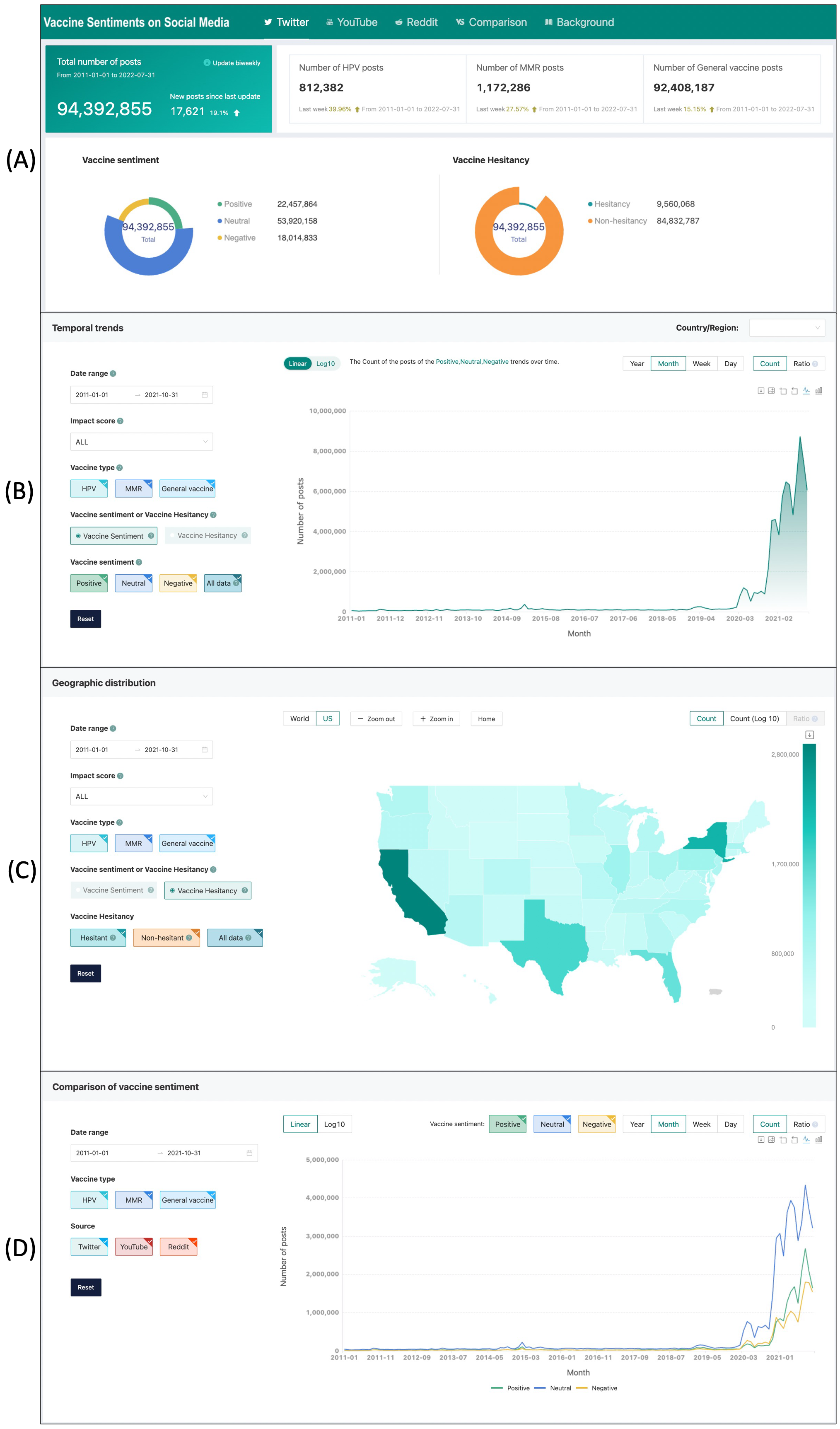


Figure S1. Dashboard screenshots of the Vaccine Sentiment and Hesitancy Trends on Social Media. (A) overview of social media discussion; (B) temporal trends of a vaccine sentiment; (C) geo-clustering of vaccine-related discussions; (D) comparison of different vaccine sentiments.


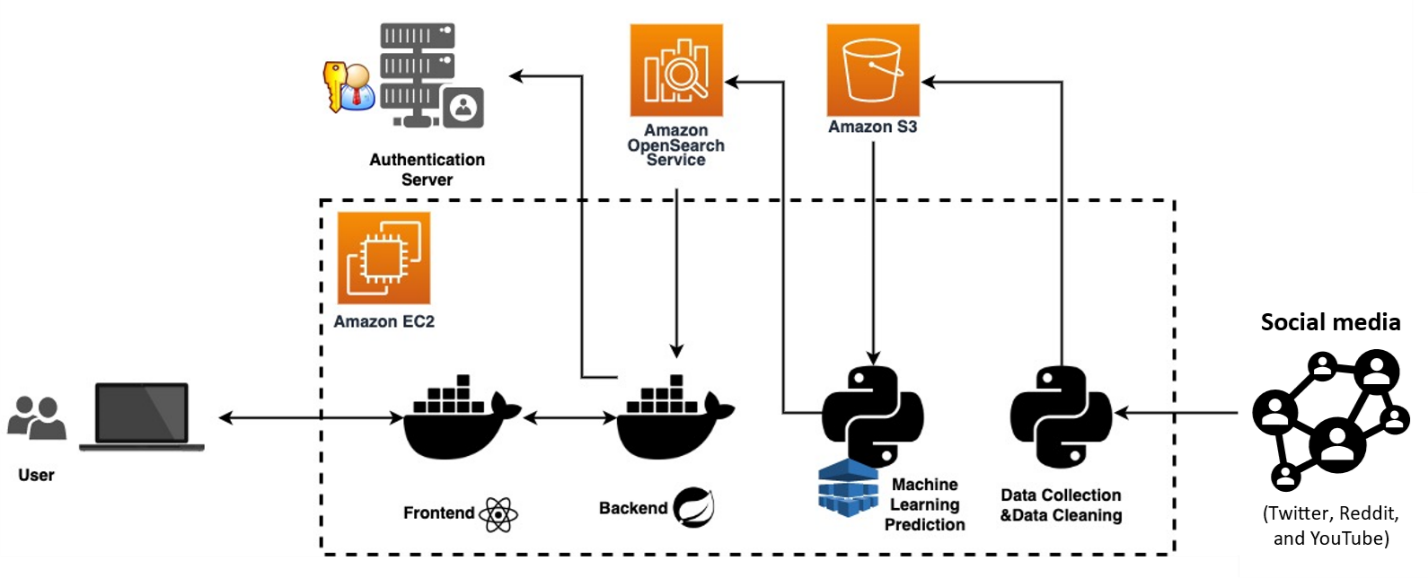


Figure S2. The system structure and data flow diagram of the interactive vaccine dashboard.

Table S1. Keywords for data collection.

| **Keyword type** | **Vaccine topic** | **Keywords** |
| --- | --- | --- |
| Searching keywords | HPV | “anal intraepithelial neoplasia”, “vaginal cancer”, “anal cancer”, “cancer anal”, “cervical”, “cancer anus”, “cancer cervical”, “cancer head neck”, “papilloma”, “cancer penile”, “cancer penis”, “cancer vagina”, “cancer vulva”, “carcinoma anal”, “carcinoma anus”, “carcinoma in situ”, “carcinoma penile”, “carcinoma penis”, “carcinoma vagina”, “carcinoma vulva”, “cervarix”, “cervical dysplasia”, “cervical intraepithelial neoplasia”, “cervical neoplas”, “gardasil”, “genita wart”, “hpv”, “hrhpv”, “hsil”, “laryngeal papillomatosis”, “lsil”, “neoplas anal”, “neoplas anus”, “neoplas penile”, “neoplas penis”, “papilloma virus”, “papillomavirus”, “papillomavirus infections”, “penile intraepithelial neoplasia”, “recurrent respiratory papillomatosis”, “tumor anal”, “tumor anus”, “vaginal dysplasia”, “vaginal intraepithelial neoplasia”, “vulvar intraepithelial neoplasia” |
|  | MMR | “mmr”, “measles”, “measle”, “mumps”, “rubella”, “mmr ii”, “m-m-r ii”, “mmrv”, “mmr v”, “proquad”, “priorix” |
|  | General | “vaccinated”, “vaccination”, “vaccine”, “vaccin”, “vax”, “inoculate”, “inoculation”, “immunize”, “immunise”, “immunization”, “immunisation” |
| Inclusion keywords | HPV | “cervarix”, “gardasil”, “inject”, “immuni”, “immunized”, “immunization”, “immunisation”, “immunity”, “vax”, “dose”, “needle”, “inoculat”, “inoculate”, “inoculation”, “inoculated”, “shot”, “vaccin”, “vaccine”, “vaccination”, “vaccinated”, “jab” |
|  | MMR | “inject”, “immuni”, “immunized”, “immunization”, “immunisation”, “immunity”, “vax”, “dose”, “needle”, “inoculat”, “inoculate”, “inoculation”, “inoculated”, “shot”, “vaccin”, “vaccine”, “vaccination”, “vaccinated”, “jab” |
|  | General | NA |
| Exclusion keywords | HPV | “heroin”, “meth “, “addict”, “needle sharing” |
|  | MMR | “heroin”, “meth “, “addict”, “needle sharing” |
|  | General | “heroin”, “meth “, “addict”, “needle sharing” |

Table S2. Illustrative annotations for social media posts. The labels of 3Cs model are highlighted in bold if they lead the posts classified as “Hesitant”.

| **Illustrative post** | **Vaccine sentiment** | **Vaccine hesitancy** | | | | |  |  |
| --- | --- | --- | --- | --- | --- | --- | --- | --- |
|  |  | **World Health Organization’s 3Cs model** | | | **Overall hesitancy** | | |  |
|  |  | **Confidence** | **Complacency** | **Convenience** | |  | | |
| “COVID vaccine is not safe, also I don’t need it to protect myself as I’ve got natural immunity.” | Negative | **Lack of confidence** | **Complacent** | Convenient | | Hesitant | | |
| “The measles is a benign disease and there is no need for vaccines.” | Negative | Confident | **Complacent** | Convenient | | Hesitant | | |
| “For now, get vaccinated to protect you in the future.” | Positive | Confident | No complacency | Convenient | | Nonhesitant | | |

Table S3. Number of annotated posts for each social media platform, categorized by vaccine sentiment and World Health Organization’s 3Cs model vaccine hesitancy group. HPV: human papillomavirus vaccines; MMR: measles, mumps, and rubella vaccines; General: general/unspecified vaccines.

| **Dataset** | | | **Sentiment** | | | **WHO’s 3Cs Models** | | | | | | | | **Hesitancy** | | |  |
| --- | --- | --- | --- | --- | --- | --- | --- | --- | --- | --- | --- | --- | --- | --- | --- | --- | --- |
|  |  |  |  |  |  | **Confidence** | | **Complacency** | | | **Convenience** | | |  |  |  |  |
| **Platform** | **Vaccine topic** | **# of posts** | **Positive** | **Neutral** | **Negative** | **Confident** | **Lack of confidence** | | **Complacent** | **No complacency** | | **Convenient** | **Inconvenient** | | **Hesitant** | **Nonhesitant** | |
| Twitter | HPV | 10,000 | 5,865 | 3,135 | 1,000 | 146 | 668 | | 97 | 717 | | 753 | 61 | | 814 | 9,186 | |
|  | MMR | 10,000 | 2,744 | 5,390 | 1,866 | 187 | 1,635 | | 172 | 1,650 | | 1,804 | 18 | | 1,822 | 8,178 | |
|  | General | 10,000 | 1,562 | 6,765 | 1,673 | 475 | 939 | | 435 | 979 | | 1,372 | 42 | | 1,414 | 8,586 | |
| Reddit | HPV | 5,000 | 2,084 | 2,345 | 571 | 61 | 268 | | 24 | 305 | | 286 | 43 | | 329 | 4,671 | |
|  | MMR | 5,000 | 1,697 | 2,559 | 744 | 270 | 308 | | 261 | 317 | | 568 | 10 | | 578 | 4,422 | |
|  | General | 5,000 | 731 | 3,907 | 362 | 54 | 245 | | 32 | 267 | | 277 | 22 | | 299 | 4,701 | |
| YouTube | HPV | 4,393 | 1,258 | 1,758 | 1,377 | 248 | 1,094 | | 212 | 1,130 | | 1,305 | 37 | | 1,342 | 3,051 | |
|  | MMR | 5,000 | 1,375 | 2,360 | 1,265 | 28 | 1,172 | | 19 | 1,181 | | 1,189 | 11 | | 1,200 | 3,800 | |
|  | General | 5,000 | 411 | 2,183 | 2,406 | 74 | 1,554 | | 71 | 1,557 | | 1,617 | 11 | | 1,628 | 3,372 | |

Table S4. Text classification model performance on vaccine sentiment. The label distribution refers to the distribution of labels in the annotated dataset. The annotated corpus for each task was split into train, valid, and test sets with a proportion of 6:2:2. HPV: human papillomavirus vaccines; MMR: measles, mumps, and rubella vaccines; General: general/unspecified vaccines; SVM: Support Vector Machine; LR: Logistic Regression.

| **Dataset** | | **Label distribution** | | | **Performance** | | | | | **Best model** |
| --- | --- | --- | --- | --- | --- | --- | --- | --- | --- | --- |
| **Platform** | **Vaccine**  **topic** | **Positive** | **Neutral** | **Negative** | **Positive**  **F1** | **Neutral**  **F1** | **Negative**  **F1** | **Accuracy** |  | |
| Twitter | HPV | 59% | 31% | 10% | 0.87 | 0.71 | 0.41 | 0.78 | SVM | |
|  | MMR | 27% | 54% | 19% | 0.57 | 0.67 | 0.53 | 0.61 | LR | |
|  | General | 16% | 68% | 17% | 0.47 | 0.83 | 0.43 | 0.73 | SVM | |
| Reddit | HPV | 42% | 47% | 11% | 0.67 | 0.67 | 0.32 | 0.63 | LR | |
|  | MMR | 34% | 51% | 15% | 0.50 | 0.65 | 0.26 | 0.55 | LR | |
|  | General | 15% | 78% | 7% | 0.35 | 0.86 | 0.21 | 0.75 | LR | |
| YouTube | HPV | 29% | 40% | 31% | 0.58 | 0.51 | 0.60 | 0.56 | LR | |
|  | MMR | 28% | 47% | 25% | 0.53 | 0.59 | 0.49 | 0.55 | LR | |
|  | General | 8% | 44% | 48% | 0.19 | 0.51 | 0.59 | 0.51 | LR | |

Table S5. Text classification model performance on vaccine hesitancy. The label distribution refers to the distribution of labels in the annotated dataset. The annotated corpus for each task was split into train, valid, and test sets with a proportion of 6:2:2. HPV: human papillomavirus vaccines; MMR: measles, mumps, and rubella vaccines; General: general/unspecified vaccines; LR: Logistic Regression; RF: Random Forest.

| **Dataset** | | **Label distribution** | | **Performance** | | | | **Best model** | |
| --- | --- | --- | --- | --- | --- | --- | --- | --- | --- |
| **Platform** | **Vaccine**  **topic** | **Hesitant** | **Nonhesitant** | **Hesitant F1** | **Nonhesitant**  **F1** | **Accuracy** |  | |  |
| Twitter | HPV | 8% | 92% | 0.40 | 0.94 | 0.90 | LR | |  |
|  | MMR | 18% | 82% | 0.44 | 0.90 | 0.83 | RF | |  |
|  | General | 14% | 86% | 0.38 | 0.89 | 0.82 | LR | |  |
| Reddit | HPV | 7% | 93% | 0.19 | 0.87 | 0.78 | Snorkel | |  |
|  | MMR | 12% | 88% | 0.23 | 0.81 | 0.69 | Snorkel | |  |
|  | General | 6% | 94% | 0.20 | 0.95 | 0.91 | LR | |  |
| YouTube | HPV | 31% | 69% | 0.58 | 0.81 | 0.73 | LR | |  |
|  | MMR | 24% | 76% | 0.53 | 0.83 | 0.75 | LR | |  |
|  | General | 33% | 67% | 0.61 | 0.76 | 0.70 | LR | |  |

Table S6. Text classification model performance on vaccine hesitancy: lack of confidence. The label distribution refers to the distribution of labels in the annotated dataset. The annotated corpus for each task was split into train, valid, and test sets with a proportion of 6:2:2. HPV: human papillomavirus vaccines; MMR: measles, mumps, and rubella vaccines; General: general/unspecified vaccines; LR: Logistic Regression; SVM: Support Vector Machine; RF: Random Forest; XGB: eXtreme Gradient Boosting.

| **Dataset** | | **Label distribution** | | **Performance** | | | **Best model** |
| --- | --- | --- | --- | --- | --- | --- | --- |
| **Platform** | **Vaccine**  **topic** | **Confident** | **Lack of confidence** | **Confident F1** | **Lack of confidence F1** | **Accuracy** |  |
| Twitter | HPV | 18% | 82% | 0.35 | 0.88 | 0.80 | LR |
|  | MMR | 10% | 90% | 0.31 | 0.95 | 0.90 | SVM |
|  | General | 34% | 66% | 0.52 | 0.79 | 0.71 | RF |
| Reddit | HPV | 19% | 81% | 0.35 | 0.86 | 0.77 | Snorkel |
|  | MMR | 47% | 53% | 0.62 | 0.74 | 0.69 | Snorkel |
|  | General | 18% | 82% | 0.56 | 0.84 | 0.77 | Snorkel |
| YouTube | HPV | 18% | 82% | 0.44 | 0.89 | 0.82 | XGB |
|  | MMR | 2% | 98% | 0.29 | 0.99 | 0.98 | LR |
|  | General | 5% | 95% | 0.63 | 0.98 | 0.95 | XGB |

Table S7. Text classification model performance on vaccine hesitancy: complacency. The label distribution refers to the distribution of labels in the annotated dataset. The annotated corpus for each task was split into train, valid, and test sets with a proportion of 6:2:2. HPV: human papillomavirus vaccines; MMR: measles, mumps, and rubella vaccines; General: general/unspecified vaccines; LR: Logistic Regression; RF: Random Forest.

| **Dataset** | | **Label distribution** | | **Performance** | | | **Best model** |
| --- | --- | --- | --- | --- | --- | --- | --- |
| **Platform** | **Vaccine**  **topic** | **Complacent** | **No complacency** | **Complacent**  **F1** | **No complacency**  **F1** | **Accuracy** |  |
| Twitter | HPV | 12% | 88% | 0.47 | 0.94 | 0.89 | Snorkel |
|  | MMR | 9% | 91% | 0.36 | 0.91 | 0.84 | LR |
|  | General | 31% | 69% | 0.41 | 0.81 | 0.71 | RF |
| Reddit | HPV | 7% | 93% | 0.43 | 0.93 | 0.88 | Snorkel |
|  | MMR | 45% | 55% | 0.68 | 0.59 | 0.64 | Snorkel |
|  | General | 11% | 89% | 0.60 | 0.96 | 0.93 | Snorkel |
| YouTube | HPV | 16% | 84% | 0.33 | 0.91 | 0.84 | RF |
|  | MMR | 2% | 98% | 0.50 | 1.00 | 0.99 | LR |
|  | General | 4% | 96% | 0.60 | 0.97 | 0.95 | LR |

Table S8. Text classification model performance on vaccine hesitancy: inconvenience. The label distribution refers to the distribution of labels in the annotated dataset. The annotated corpus for each task was split into train, valid, and test sets with a proportion of 6:2:2. HPV: human papillomavirus vaccines; MMR: measles, mumps, and rubella vaccines; General: general/unspecified vaccines; XGB: eXtreme Gradient Boosting.

| **Dataset** | | **Label distribution** | | **Performance** | | | **Best model** |
| --- | --- | --- | --- | --- | --- | --- | --- |
| **Platform** | **Vaccine**  **topic** | **Convenient** | **Inconvenient** | **Convenient F1** | **Inconvenient F1** | **Accuracy** |  |
| Twitter | HPV | 93% | 7% | 0.96 | 0.48 | 0.92 | Snorkel |
|  | MMR | 99% | 1% | 0.99 | 0.18 | 0.98 | Snorkel |
|  | General | 97% | 3% | 0.99 | 0.55 | 0.98 | Snorkel |
| Reddit | HPV | 87% | 13% | 0.94 | 0.67 | 0.89 | Snorkel |
|  | MMR | 98% | 2% | 0.95 | 0.17 | 0.91 | Snorkel |
|  | General | 93% | 7% | 0.98 | 0.50 | 0.97 | Snorkel |
| YouTube | HPV | 97% | 3% | 0.98 | 0.17 | 0.96 | XGB |
|  | MMR | 99% | 1% | 1.00 | 0.50 | 0.99 | Snorkel |
|  | General | 99% | 1% | 0.99 | 0.20 | 0.98 | Snorkel |
